# Neutrophil Gelatinase-Associated Lipocalin as a Biomarker in Post-Acute COVID-19 Syndrome

**DOI:** 10.3390/jcm13071851

**Published:** 2024-03-23

**Authors:** Karol Żmudka, Alicja Gałeczka-Turkiewicz, Aleksandra Wroniecka, Aleksandra Włosowicz, Barbara Sobala-Szczygieł, Jolanta Mrochem-Kwarciak, Zenon P. Czuba, Jerzy Jaroszewicz

**Affiliations:** 1 Department of Infectious Diseases and Hepatology, Medical University of Silesia in Katowice, 41-902 Bytom, Poland; s78586@365.sum.edu.pl (K.Ż.); s73510@365.sum.edu.pl (A.G.-T.); s74351@365.sum.edu.pl (A.W.); s71971@365.sum.edu.pl (A.W.); sobala.szczygiel@op.pl (B.S.-S.); 2 Analytics and Clinical Biochemistry Department, Maria Sklodowska-Curie National Research Institute of Oncology, Gliwice Branch, 44-102 Gliwice, Poland; jolanta.mrochem-kwarciak@gliwice.nio.gov.pl; 3 Department of Microbiology and Immunology, Faculty of Medical Sciences in Zabrze, Medical University of Silesia in Katowice, Jordana 19, 41-808 Zabrze, Poland; zczuba@sum.edu.pl

**Keywords:** SARS-CoV-2, COVID-19, NGAL, post-acute COVID-19 syndrome

## Abstract

**Background**: Neutrophil gelatinase-associated lipocalin (NGAL) is part of the innate immune system and acute-phase protein. Current data state that acute COVID-19 patients have higher levels of serum NGAL (sNGAL), but it is not known if higher protein levels are maintained in the convalescents. As post-COVID complications are currently the most important aspect of the disease, further research into metabolic and immunological consequences of the disease is needed. **Methods**: We aimed to determine the levels of sNGAL in a patient population 3 months after the acute phase of the disease and to identify the factors that may be related to the elevation of sNGAL levels in the mentioned cohort. The study included 146 patients diagnosed with COVID-19 in different stages of the disease. Three months after COVID-19 diagnosis, patients’ sera were sampled and tested. **Results**: We demonstrate an association between the severity of the disease in the acute phase and elevated sNGAL levels three months after recovery, with the exception of the most severe hospitalized patients, who received early treatment. Moreover, we establish that sNGAL levels could be associated with prolonged dyspnea and the regulation of hunger and satiety in COVID-19 convalescents. **Conclusions**: These observations support the view that the introduction of antiviral treatment, steroids, and intense oxygen therapy reduces post-COVID immune-associated complications.

## 1. Introduction

Despite the introduction of prophylactic vaccines, COVID-19 disease remains one of the most current epidemiological problems in global health care. Moreover, the long-term effects of the disease on convalescents are the main health concern after the pandemic. The COVID-19 disease is caused by severe acute respiratory syndrome coronavirus 2 (SARS-CoV-2) that primarily infects the respiratory system and causes multiorgan changes by inducing cell death and the activation of secretion of proinflammatory cytokines [1]. The clinical spectrum of COVID-19 disease ranges from asymptomatic to fatal infections. Moreover, some patients can experience persistent symptoms and end-organ dysfunction [2]. The World Health Organization defines long-COVID as a ‘condition that occurs in individuals with a history of probable or confirmed SARS-CoV-2 infection, usually 3 months from the onset, with symptoms that last for at least 2 months and cannot be explained by an alternative diagnosis’ [3]. The main symptoms of post-acute COVID-19 syndrome (PACS) are fatigue, headache, attention disorder, and dyspnea, but other effects include abnormalities in the respiratory, cardiovascular, and nervous systems [4,5,6]. Interestingly, it has been observed that post-COVID-19 syndrome is more common not only in patients with severe SARS-CoV-2 infection but also in those who have not been vaccinated [7].

Neutrophil gelatinase-associated lipocalin (NGAL), also known as lipocalin-2, is part of the innate immune system and is an acute-phase protein [8,9]. Since it is rapidly discharged in response to tubular damage, urinary NGAL (uNGAL) is used as an early diagnostic biomarker for kidney injury [10]. It has been shown that SARS-CoV-2 infection can result in acute kidney injury (AKI) in up to 25% of patients, especially those with diabetes, hypertension, or chronic kidney disease. The pathogenesis of kidney impairment in COVID-19 is multifactorial and may include virus interaction with the angiotensin-converting enzyme 2 receptor (highly expressed in the kidneys), endothelial and immune dysfunction, coagulopathy, and complement activation [1,11]. Importantly, NGAL is a protein secreted not only in the kidney but also in the lung, pancreas, intestine, thymus, and cancerous tissues (colorectal, lung, and pancreatic adenocarcinoma) [12]. Considering that lung injury can occur during COVID-19, it is significant that lung epithelial damage is associated with elevated serum NGAL (sNGAL) levels. It was demonstrated that increased sNGAL can be a biomarker of pulmonary injuries in mechanically ventilated patients and can be associated with early mortality in patients with ARDS [13,14]. Physiologically, NGAL is present in the bloodstream at very low levels, which can increase significantly in the event of ischemic or toxic injury. Serum NGAL (sNGAL) is filtered by glomeruli and then reabsorbed by proximal tubules [13]. Thus, lung epithelial damage results in elevated sNGAL levels. The aim of this study is to evaluate the role of sNGAL measured in COVID-19 convalescents as a biomarker of persistent inflammation.

## 2. Materials and Methods

The study included 146 adult patients who were diagnosed with COVID-19 by a reverse transcription polymerase chain reaction (RT-PCR) test from nasopharyngeal swabs. Testing was conducted from mid to late 2020 including the first wave of COVID-19 disease in Poland and thus presumably included mostly the Alpha variant of the virus. Those who tested positive were invited to voluntarily participate in the trial. The Bioethical Committee of the Medical University of Silesia in Katowice approved the study (17/2020, 1 June 2020).

The severity of the disease was measured with a modified WHO scale [15]. Group 1 were asymptomatic or sparsely symptomatic patients presenting with symptoms such as fever, myalgia, vomiting, nausea, or diarrhea. Group 2 included patients with exhaustion, asthenia, fever above 38 degrees Celsius, cough and dyspnea, and other symptoms but without features of respiratory failure. Patients in Group 3 had clinical and laboratory features of respiratory deterioration with dyspnea and the presence of tachypnoea but were not hospitalized. The 4th group consisted of hospitalized patients with acute respiratory distress syndrome (ARDS) who, in addition, had failure of other organs. The last group of patients received in-hospital treatment, which included steroids, antiviral treatment, and oxygen therapy. Patients in Groups 1, 2, and 3, who were not hospitalized, did not receive such treatment, and we presumed that those patients only used over-the-counter drugs.

Three months after the acute phase of the disease, patients were interviewed about the selected symptoms of COVID-19 that were present during or shortly after the acute phase of the disease and their pre-existing comorbidities. Moreover, participants were asked about the current (3 months after the acute phase of the disease) presence of dyspnea, sleep, or cognitive disorders. Later the same day, blood samples were obtained from the study participants. Sample collection was conducted under standard conditions in the Department of Infectious Diseases and Hepatology of the Medical University of Silesia, Katowice. The patients were in a fasting state. Blood samples were collected between 7.00 and 9.00 o’clock, using a vacuum Becton–Dickinson system to sample tubes without anticoagulant. Later, after centrifugation at 3000 rotations/min for 10 min at a temperature of 40 °C, samples were frozen at −20 °C. Those samples were then used to quantify levels of sNGAL expression using a Chemiluminescent Microparticle Immunoassay 09P29 Alinity I Urine NGAL Reagent Kit in The Maria Sklodowska-Curie National Research Institute of Oncology in Gliwice. Moreover, the quantification of the following cytokine concentrations was conducted: Cytokine concentrations (IL-1b, IL-1ra, IL-2, IL-4, IL-5, IL-6, IL-7, L-8, IL-9, IL-10, IL-12p70, IL-13, IL-15, IL-17A, eotaxin, FGF basic, G-CSF, GM-CSF, IFN-γ, IP-10, MCP-1, MIP-1a, MIP-1b, PDGF-bb, RANTES, TNF-α, and VEGF) in serum were measured by the Bio-Plex Human Cytokine Panel 27-Plex (Bio-Rad Laboratories Inc., Hercules, CA, USA) according to the manufacturer’s instructions. The assay was performed using the Bio-Plex^TM^ 200 System and Bio-Plex Manager 6.2 software (Bio-Rad Laboratories Inc., USA). The intra-assay %CV varied up to 15%, and the inter-assay %CV varied up to 25%, depending on the analyzed cytokine. The method has been previously used in measuring cytokines in serum and supernatants [16,17].

The blood specimens were also tested for the following:

- Biochemical markers (ALT, AST, bilirubin, creatinine, ferritin, GGTP, albumins, gamma globulins, INR, D-dimers, CRP, and HbA1c);
- White blood cell, monocyte, and neutrophil count.

Lastly, 9 months after the acute phase of the disease, telephone follow-up was conducted to determine the presence of dyspnea, sleep, or cognitive disorder at the time point of the follow-up. Patients with any symptoms of PACS were subdivided into groups according to the reported presence of dyspnea, sleep, or cognitive disorder. We performed nonparametric analysis with the Mann–Whitney U test to determine if sNGAL levels were significantly different in certain subgroups. We aimed to determine if sNGAL levels were associated with the presence of any of the three main PACS symptoms. Additionally, we investigated whether sNGAL levels were linked to one specific PACS complication. Lastly, we explored the association between sNGAL levels and the reported duration of a symptom. A detailed description of the methodology is presented in Appendix A. We performed additional analysis to identify predictors for persistent dyspnea at the 9-month follow-up call. 

We report medians and interquartile ranges (IQRs) for nonparametric continuous variables and proportions for categorical variables. To compare data between two non-normally distributed independent groups, we used the Mann–Whitney U test, and for three or more independent groups, we used the Kruskal–Wallis test. For all analyses, two-sided *p* values ≤ 0.05 were considered statistically significant. All statistical analysis was performed using RStudio statistical software - 2022.07.2 Build 576 (R Studio Inc., Boston, MA, USA). 

## 3. Results

### 3.1. General Characteristics of Studied Population

Out of the 149 participants included in the analysis, 94 were females (64%), and 54 (36%) were males, and the median age was 49 years (IQR: 41.5–57.5). Among the 122 nonhospitalized patients, 23 were categorized as Group 1 for COVID-19 severity, 77 as Group 2, and 22 as Group 3. Only 24 patients were hospitalized and, consequently, were included in Group 4 of the COVID-19 severity scale. No patient died during the course of the trial, and none resigned during it. Therefore, all patients who initially agreed to take part in the trial were included in the analysis. As mentioned before, NGAL is a well-established marker of acute kidney injury; thus, we also analyzed patients for potential kidney disease. In the case of the studied population, all participants had creatinine levels within the reference range with a median of 64.9 (IQR: 57–74.5) µmol/L. Moreover, the patients were asked about pre-existing diseases, including renal diseases. Only one patient declared well-controlled chronic kidney disease Below we present characterization of comorbidities and symptoms present in the studied population (Table 1).

### 3.2. Symptoms, Comorbidities, COVID-19 Severity, and sNGAL Levels

**Figure 1:**
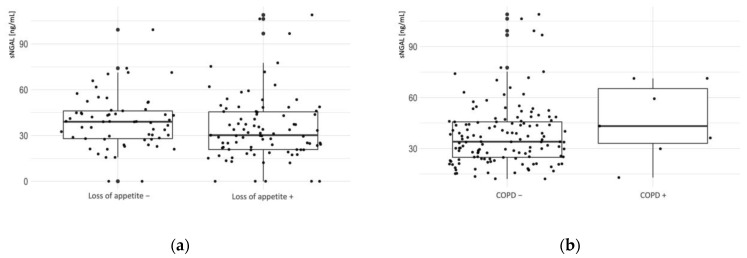

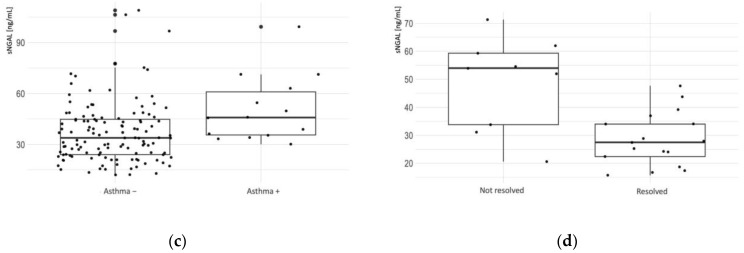
Boxplots presenting sNGAL levels among all patients by (**a**) asthma, with (+) meaning sNGAL levels in patients with asthma and (–) meaning sNGAL levels in patients without asthma; (**b**) COPD, with (+) meaning sNGAL levels in patients with COPD and (–) meaning sNGAL levels in patients without COPD; (**c**) reduced appetite, with (+) meaning sNGAL levels in patients who reported reduced appetite and (–) meaning sNGAL levels in patients who did not report reduced appetite; (**d**) a boxplot of sNGAL concentration in patients for whom dyspnea resolved or not between the 3rd and 9th month after the infection.

The distribution of sNGAL significantly deviated from normality based on the Shapiro–Wilk test results (W = 0.898, *p* < 0.001). We determined the median level of sNGAL as 35.0 ng/mL (IQR = 25.0–46.0) and did not find significant differences in the distribution of sNGAL between male and female patients. In order to assess whether any mentioned comorbidities or symptoms were associated with higher sNGAL levels in the studied population, we performed the Mann–Whitney U test between those who had a factor present and those who did not (Table 2). Among the reported comorbidities, we found that only patients suffering from asthma and COPD (chronic obstructive pulmonary disease) had significantly higher levels of sNGAL (Figure 1). Comparing sNGAL levels of participants who experienced specific symptoms with those who did not experience those symptoms, we found that convalescents with reduced appetite had statistically significantly lower levels of sNGAL.

**Figure 2:**
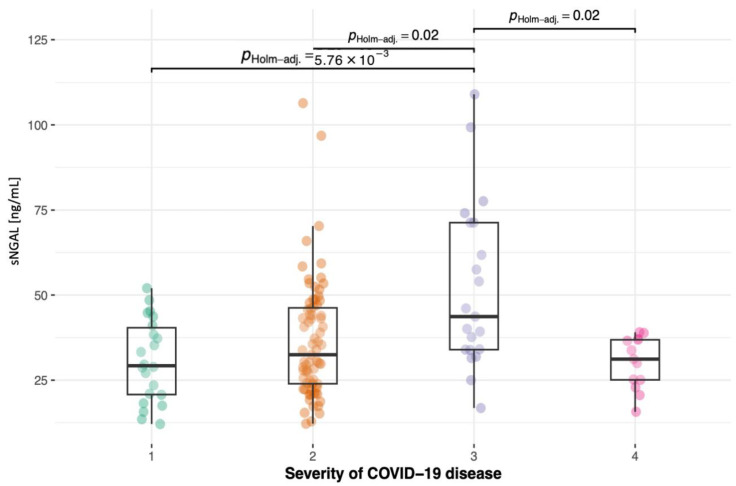
Boxplot depicts sNGAL levels in the groups according to the modified WHO COVID-19 severity scale presented above. Serum NGAL levels were significantly higher in the group of nonhospitalized patients at the 3rd stage, whereas 4th-stage patients who were hospitalized and received treatment presented lower sNGAL levels; *p* values for each pair are presented.

As mentioned above, all patients were assigned to one of the four groups in the modified COVID-19 disease severity scale by the WHO. Data across the four groups were compared using the Kruskal–Wallis test to assess the differences in sNGAL levels among the groups (Figure 2). We found that sNGAL levels were highest in the third group of COVID-19 severity. Interestingly, the hospitalized patients, who were most affected by the disease, did not present significantly higher sNGAL levels than the first and second groups.

**Figure 3:**
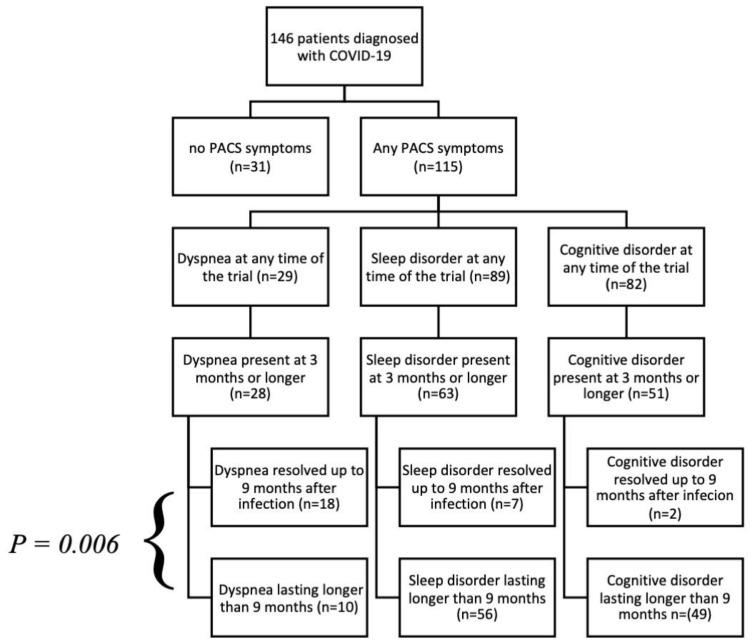
The chart presents the number of patients in groups having certain symptoms of PACS. sNGAL levels between groups were compared according to the methodology presented above. The only significant sNGAL level difference is depicted with *p*-value—comparison of sNGAL levels between groups with dyspnea resolving up to 9 months after the infection and those with dyspnea lasting longer than 9 months.

When analyzing the symptoms reported 3 to 9 months after the acute phase, we found that participants who had dyspnea that resolved between the 3rd and 9th month after the infection had significantly lower sNGAL levels than those who had persistent dyspnea for more than 9 months (*p* = 0.006) (Figure 1d) and (Figure 3). 

Logistic regression was used to analyze the correlation between the presence of dyspnea lasting longer than 9 months and the presence of asthma, COPD, appetite loss, and stage 3 of COVID-19 disease (Figure 3). It was found that the odds of dyspnea lasting longer than 9 months increased significantly with the presence of asthma ([OR] = 4.43, 95% CI [0.87, 18.38], *p* = 0.048) and COPD ([OR] = 6.55, 95% CI [0.85, 36.26], *p* = 0.039) while being insignificant for appetite loss and stage 3 COVID-19 disease (*p* > 0.05).

### 3.3. The Correlation of Blood Specimen Results with sNGAL Levels

**Figure 4:**
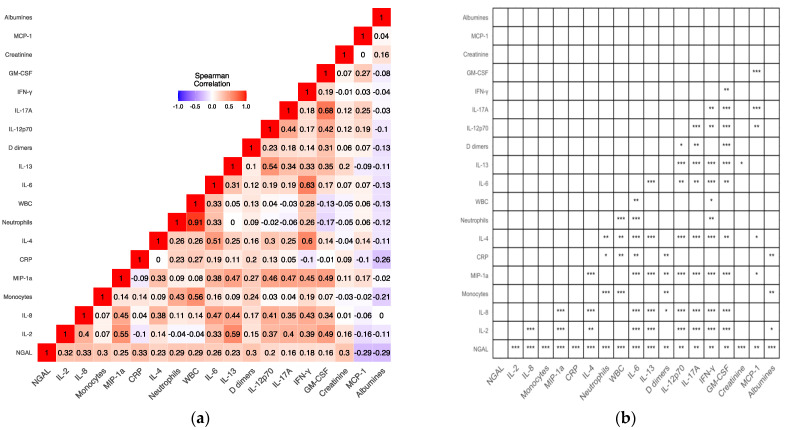
Matrices present the results of the Spearman correlation analysis of the factors mentioned above. Heatmap (**a**) presents correlation coefficients and matrix (**b**) depicts the associated *p*-values for each correlation coefficient (* for *p* ≤ 0.05, ** for *p* ≤ 0.01, *** *p* ≤ 0.001). Only factors correlating with NGAL above r = 0.2 are included. IL, interleukin; MCP-1, monocyte chemoattractant protein 1; GM-CSF, granulocyte–macrophage colony-stimulating factor; IFN-γ, interferon-gamma; WBC, white blood cells; CRP, C-reactive protein; MIP, macrophage inflammatory protein.

Spearman’s rank correlation was computed to determine the association between sNGAL levels and cytokine expression; biochemical markers; and white blood cell, monocyte, and neutrophil levels (Figure 4).. Below, we present the median values and interquartile ranges of the analyzed markers: Il-2—0.595 (IQR: 0.11–0.73) pg/mL; Il-8—8.71 (IQR: 6.68–11.55) pg/mL; monocytes—0.5 *×* 10^9^/L (IQR: 0.4 *×* 10^9^/L—0.64 *×* 10^9^/L); MIP-1a—1.19 (IQR: 0.77–1.59) pg/mL; CRP—1 (IQR: 0.5–2.17) mg/dL; Il-4—2.13 (IQR: 1.85–2.37) pg/mL; neutrophils—3.42 *×* 10^9^/L (IQR: 2.7 *×* 10^9^/L–4.3 *×* 10^9^/L); WBC—6.1 *×* 10^9^/L (IQR: 5.13 *×* 10^9^/L–7.3 *×* 10^9^/L); Il-6—3.95 (IQR: 2.41–6.14) pg/mL); Il-13—1.58 (IQR: 1.02–6.35) pg/mL; D-dimers—282.5 (IQR: 222–373) µg/L; Il-12p70—3.59 (IQR: 2.03–5.31) pg/mL; Il-17A—3.02 (IQR: 1.65–4.82) pg/mL; IFN-γ—80.8 (IQR: 62.03–100.4) pg/mL; GM-CSF—5.08 (IQR: 4.15–10.09) pg/mL; creatinine—65.27 (IQR: 57.27–74.68) µmol/L; MCP-1—4.11 (IQR: 1.11–13.15) pg/mL; and albumins—43 (IQR: 34–54) g/L. We did not find any strong correlation between sNGAL levels and other tested factors. We found that sNGAL weakly correlated with both proinflammatory (IL-12, IFN-γ, and GM-CSF) and anti-inflammatory cytokines (IL-4) and other either anti-inflammatory or proinflammatory cytokines such as IL-6.

## 4. Discussion

NGAL, also known as lipocalin-2, is a protein whose diagnostic features are still being explored. It is characterized by its high production in neutrophils, and its expression is also present in tissues throughout the body, including the lungs, kidneys, heart, intestines, and adipose tissue. It has antibacterial effects as, by participating in iron sequestration, it prevents iron use by these microorganisms. As a marker, it is mainly known for its importance in the early diagnosis of acute kidney injury. However, due to its wide distribution in the body, it is assumed to be an important indicator of many diseases [18]. Recovering from COVID-19 may be complicated by developing PACS symptoms such as fatigue, dyspnea, or gastrointestinal problems. Therefore, NGAL, which is a recognized marker of inflammation, is associated with these symptoms and can be potentially used to monitor patients’ status. In a prospective cohort study involving adult patients recovering from acute COVID-19 pneumonia, researchers assessed clinical variables during 3- and 6-month follow-up visits. The study concluded that these follow-up visits, which included chest computed tomography (CT) scans and pulmonary function tests (PFTs), showed limited utility as most clinical variables did not exhibit significant changes [19]. Considering the apparent lack of necessity for CT scans and PFTs, the measurement of sNGAL levels, which may be linked to persistent dyspnea, can serve as a promising marker for identifying persistent dyspnea in some patients. 

In our study, the aim was to assess the sNGAL concentration in patients who were hospitalized due to SARS-CoV-2 infection. COVID-19 is a disease with a characteristic exaggerated immune response—the cytokine storm [20,21]. NGAL has a multifunctional role in regulating cytokine levels. The literature reports that activating/stimulating interleukins like IL-6 and IL-2 are regulators of NGAL concentration, which may confirm the role of NGAL as an acute-phase protein [22,23,24]. However, a paper by Buonafine et al. stated that NGAL is capable of stimulating proinflammatory cytokine production and can initiate the inflammatory process autonomously [25]. On the other hand, research by Guo et al. suggests that NGAL can be an anti-inflammatory modulator in macrophage activation [26]. Based on this information, NGAL can be cautiously used as a marker to detect and monitor more severely ill patients with a stronger immune response. Our study demonstrated that the severity of COVID-19 may be associated with increased sNGAL concentration three months after recovery. Additionally, a correlation was observed between NGAL and IL-6, IL-12, IFN-γ, and GM-CSF—proinflammatory cytokines—and strongly chemotactic IL-8 in patients suffering from severe COVID-19 [27]. Moreover, the role of IL-8 in the induction of prothrombotic effects was described by Kaiser et al., stating that IL-8 enhances neutrophil extracellular trap (NET) formation and the activation of the coagulation process, which is responsible for thromboembolic complications in COVID-19 patients [28].

Our research showed the highest concentration of sNGAL among nonhospitalized patients diagnosed with the stage 3 severity of COVID-19, which included patients with clinical and laboratory features of respiratory deterioration (e.g., dyspnea or tachypnoea) but not hospitalized. The study carried out by Can et al. among pregnant women showed that a group of hospitalized pregnant women with more severe symptoms had higher sNGAL levels than those with less severe COVID-19 infection [29]. However, in the mentioned study, all pregnant patients were hospitalized and were divided only into two groups. In the case of our study, we used four stages of COVID-19 severity, and most importantly, only patients in the fourth stage were hospitalized and received treatment. Due to this subdivision, we found that among the severe cases (third and fourth stage), only those who did not receive treatment had higher sNGAL levels. Hospitalized patients (fourth stage) received treatment with antiviral drugs, oxygen, and dexamethasone—an anti-inflammatory and immunosuppressive steroid that inhibits neutrophils in the course of COVID-19 [30]. As NGAL is largely produced by neutrophils, dexamethasone treatment may contribute to lower sNGAL values in hospitalized patients. On the other hand, oxygen therapy, which is used to reduce hypoxia, a known factor for multiple organ damage, could also reduce sNGAL levels.

The main symptoms of COVID-19 reported by our patients were pyrexia, fatigue, myalgia, and arthralgia, as well as loss of appetite. We found that the first four symptoms mentioned above were not associated with sNGAL concentrations. These results may be related to the fact that the assessment of sNGAL levels was carried out after the end of the acute phase of COVID-19. Furthermore, among patients who did not report loss of appetite, the sNGAL concentration was higher than among patients who reported this symptom. The reason for this phenomenon has not been substantiated in the literature and requires further investigation. 

It is commonly known that pre-existing chronic diseases in patients are related to a worse prognosis. More than 80% of the patients included in this study reported at least one comorbidity such as malignant neoplasm, diabetes, heart failure, or lung disease. Among these diseases, the group of patients with asthma and COPD had significantly higher sNGAL levels three months after COVID-19. This may be explained by the increased expression of sNGAL in the lungs of patients with COPD compared to the lungs of healthy individuals, which was confirmed in the study by Wang Y et al. [31]. In addition, a research study by Kawagoe et al. showed that sNGAL may be a marker of persistent airway obstruction among asthmatic patients, which corresponds to our findings [32]. Thus, elevated sNGAL levels among patients with COPD and asthma compared to others participating in the study do not appear to be associated with infection. 

The presence of PACS symptoms, such as dyspnea, sleep disturbances, and cognitive impairment, appeared to have no significant effect on sNGAL levels, except for those participants whose dyspnea resolved after 3 months, compared to those who had dyspnea persisting for up to 9 months. As previously mentioned, sNGAL can be affected by renal injury, airway obstruction, and many more factors; thus, it is difficult to attribute our observation to a specific one. Such observation could also be attributed to higher odds of patients having COPD and asthma in the group of patients who reported dyspnea 3 to 9 months after the acute phase of COVID-19. In the univariable logistical regression model, we observed higher odds of patients having asthma or COPD among those who reported dyspnea for 9 months or longer. However, due to the low number of cases, it is difficult to determine if the higher sNGAL levels observed are a result of the overrepresentation of patients suffering from pre-existing obstructive pulmonary diseases or if it is associated with post-COVID-19 complications. In both cases, damage to respiratory epithelial cells as a consequence of inflammation can cause the elevation of sNGAL. In another research study, it was established that mice infected with lethal doses of SARS-CoV-2 showed high viral titers in the brain and lungs with an absence in the kidneys [33]. Mice that had variable degrees of kidney proximal tubular injury displayed increased NGAL staining. This and the fact that patients with long-lasting dyspnea had higher levels of sNGAL support the thesis that increased sNGAL could be associated with persistent pulmonary epithelial damage in the course of post-COVID-19 disease. Further research is needed to explore the association of sNGAL with persistent dyspnea among patients experiencing such complications after COVID-19.

This study has potential limitations. First and foremost, it was not determined whether elevated sNGAL levels persisted throughout the three-month period following SARS-CoV-2 infection. Furthermore, our study showed that patients with loss of appetite had lower sNGAL levels than those with normal appetite, which we cannot explain after reviewing the literature. Participants were recruited from a group of patients who tested positive for COVID-19 at a single clinical testing site, thereby introducing potential selection bias. Symptoms present during the acute phase were reported to medical professionals three months later, resulting in recall bias. Moreover, the intensity of the symptoms was not measured using any standardized scale. Thus, the results based on self-reported symptoms should be considered preliminary. Further research is needed to explore the relationship between the presented symptoms and sNGAL levels in post-acute COVID-19 by employing objective measures for these symptoms. Additionally, it is important to acknowledge our limited knowledge regarding the medications administered to patients who were treated at home. We presumed that only over-the-counter medications were used in that group. Lastly, it was difficult to assess whether elevated sNGAL levels were associated with dyspnea lasting up to 9 months after the acute phase of the disease or if it was strictly associated with the increased representation of COPD and asthmatic patients in that subgroup, due to the low number of cases. 

## 5. Conclusions

To conclude, our study showed a correlation between the severity of SARS-CoV-2 infection and the elevation of sNGAL concentrations three months after the acute phase of the disease. In addition, elevated sNGAL levels in recovered patients may be associated with the occurrence of dyspnea. Importantly, the lack of a significant increase in sNGAL levels in patients who were hospitalized during a severe course of the acute phase of COVID-19 may have been due to the early administration of steroids, antiviral drugs, and oxygen therapy, as these were most often used therapeutics in hospitalized patients. Concentrations of sNGAL may also be associated with the prolonged dysregulation of hunger and satiety among patients who recovered from COVID-19.

## Figures and Tables

**Table 1:** Characterization of the study population. Number of participants with and without a comorbidity with a percentage of the total study population are presented (IQR, interquartile range; COPD, chronic obstructive pulmonary disease).

Comorbidities	No. of Participants with a Comorbidity	% in Study Population	No. of Participants without a Comorbidity	% in Study Population
Arterial hypertension	44	30.1%	102	69.9%
Myocardial infarction	17	11.6%	129	88.4%
Asthma	15	10.3%	131	89.7%
Diabetes mellitus	12	8.2%	134	91.8%
Autoimmune disease	9	6.2%	137	93.8%
Neoplasm	8	5.5%	138	94.5%
COPD	7	4.8%	139	95.2%
Heart failure	6	4.1%	140	95.9%
Venous thromboembolism	3	2.1%	143	97.9%
Viral disease	3	2.1%	143	97.9%
**Symptoms**	**No. of Participants with a Symptom**	**% in Study Population**	**No. of Participants without a Symptom**	**% in Study Population**
Fever	115	78.8%	31	21.2%
Fatigue	110	75.3%	36	24.7%
Muscle and joint pains	93	63.7%	53	36.3%
Appetite loss	87	59.6%	59	40.4%
Cough	85	58.2%	61	41.8%
Disturbances of smell	69	47.3%	77	52.7%
Nausea and sickness	58	39.7%	88	60.3%
Headache	54	37.0%	92	63.0%
Dyspnea	51	34.9%	95	65.1%
Nasal obstruction	50	34.2%	96	65.8%
Diarrhea	42	28.8%	104	71.2%
Sore Throat	39	26.7%	107	73.3%

**Table 2:** Comparisons of sNGAL levels in subgroups of patients who had or did not have certain comorbidities or symptoms. Symptoms are reported by patients during the period or shortly after the acute phase of the disease. Bold values denote statistical significance at the *p* ≤ 0.05 (IQR, interquartile range; COPD, chronic obstructive pulmonary disease).

Comorbidities	Median (IQR) Value of sNGAL without Comorbidity	Median (IQR) Value of sNGAL with Comorbidity	*p* Value
Arterial hypertension	40.1 (29.6–52.4)	33.9 (23.9–45.5)	0.069
Myocardial infarction	27.9 (24.0–43.7)	36 (25.2–46.1)	0.328
Asthma	45.9 (35.6–61)	33.9 (24–44.8)	**0.006**
Diabetes mellitus	35.4 (25.2–46.3)	32.2 (22.4–40.3)	0.412
Autoimmune disease	33.8 (27.5–51.6)	35.5 (25–45.9)	0.925
Neoplasm	38.9 (29–47.3)	35.4 (25–45.8)	0.567
COPD	51.6 (45.8–63)	34 (24.7–45.4)	**0.005**
Heart failure	31 (27.6–37.7)	35.6 (25–46.1)	0.608
Venous thromboembolism	34 (25.4–44.3)	35.5 (25.1–46)	0.834
Viral disease	46 (37–58.7)	35.4 (25–45.8)	0.328
**Symptoms**	**Median (IQR) Value of sNGAL with Certain Symptoms**	**Median (IQR) Value of sNGAL without Certain Symptoms**	***p* Value**
Fever	34.1 (25.1–45.2)	43.2 (25–52)	0.238
Fatigue	34.9 (25.5–46.5)	35.4 (23.2–44.7)	0.503
Muscle and joint pains	34 (27.5–47.3)	36 (23.4–45.4)	0.508
Appetite loss	31.7 (22.7–45.7)	39.2 (29.6–46.5)	**0.032**
Cough	36.3 (27.2–47.3)	35.3 (22.9–44.8)	0.365
Disturbances of smell	36.9 (23.9–45.7)	34.7 (26–47)	0.713
Nausea and sickness	30.8 (23.5–45)	37.3 (27.3–46.8)	0.250
Headache	36.9 (28.8–47.3)	34.1 (23.5–45.7)	0.314
Dyspnea	33.3 (25.2–45.9)	36.5 (24.7–46.2)	0.727
Nasal obstruction	36.3 (27.5–48.4)	34.7 (24–45.9)	0.613
Diarrhea	37.1 (27.5–48.5)	34.1 (24.4–44.8)	0.336
Sore Throat	38.3 (26.9–51.8)	35.3 (24.5–45.5)	0.435

## Data Availability

The data presented in this study are available upon request from the corresponding author.

## Appendix A

Patients with some symptoms of PACS were subdivided into groups according to Figure 3. We compared sNGAL levels using the Mann–Whitney U test in the following groups:

- Those who had no PACS symptoms at all vs. those who had any of the PACS symptoms;
- Those who had one of three symptoms at any time of the trial vs. those who did not develop that symptom at all;
- Those who had a certain symptom that resolved between the 3rd and 9th month vs. those for whom the symptom did not resolve and was reported to be present for more than 9 months.

No statistically significant differences in sNGAL levels were observed between the selected groups, with the exception of participants who had dyspnoea that resolved between the 3rd and 9th month after the infection compared to those who had it for more than 9 months (*p* = 0.006).

## References

[1] Serwin N., Cecerska-Heryć E., Pius-Sadowska E., Serwin K., Niedźwiedź A., Wiśniewska M., Roszak M., Grygorcewicz B., Skwirczyńska E., Machaliński B. (2022). Renal and Inflammation Markers-Renalase, Cystatin C, and NGAL Levels in Asymptomatic and Symptomatic SARS-CoV-2 Infection in a One-Month Follow-Up Study. Diagnostics.

[2] Del Rio C., Omer S.B., Malani P.N. (2022). Winter of Omicron-The Evolving COVID-19 Pandemic. JAMA.

[3] Soriano J.B., Murthy S., Marshall J.C., Relan P., Diaz J.V., WHO Clinical Case Definition Working Group on Post-COVID-19 Condition (2022). A Clinical Case Definition of Post-COVID-19 Condition by a Delphi Consensus. Lancet Infect. Dis..

[4] Lopez-Leon S., Wegman-Ostrosky T., Perelman C., Sepulveda R., Rebolledo P.A., Cuapio A., Villapol S. (2021). More than 50 Long-Term Effects of COVID-19: A Systematic Review and Meta-Analysis. Sci. Rep..

[5] Zhang N.-H., Cheng Y.-C., Luo R., Zhang C.-X., Ge S.-W., Xu G. (2021). Recovery of New-Onset Kidney Disease in COVID-19 Patients Discharged from Hospital. BMC Infect. Dis..

[6] Nalbandian A., Sehgal K., Gupta A., Madhavan M.V., McGroder C., Stevens J.S., Cook J.R., Nordvig A.S., Shalev D., Sehrawat T.S. (2021). Post-Acute COVID-19 Syndrome. Nat. Med..

[7] Schiffl H., Lang S.M. (2023). Long-Term Interplay between COVID-19 and Chronic Kidney Disease. Int. Urol. Nephrol..

[8] Nasioudis D., Witkin S.S. (2015). Neutrophil Gelatinase-Associated Lipocalin and Innate Immune Responses to Bacterial Infections. Med. Microbiol. Immunol..

[9] Bolignano D., Donato V., Coppolino G., Campo S., Buemi A., Lacquaniti A., Buemi M. (2008). Neutrophil Gelatinase-Associated Lipocalin (NGAL) as a Marker of Kidney Damage. Am. J. Kidney Dis..

[10] Bagińska J., Korzeniecka-Kozerska A. (2021). Are Tubular Injury Markers NGAL and KIM-1 Useful in Pediatric Neurogenic Bladder?. J. Clin. Med..

[11] Menez S., Parikh C.R. (2022). COVID-19 and the Kidney: Recent Advances and Controversies. Semin. Nephrol..

[12] Friedl A., Stoesz S.P., Buckley P., Gould M.N. (1999). Neutrophil Gelatinase-Associated Lipocalin in Normal and Neoplastic Human Tissues. Cell Type-Specific Pattern of Expression. Histochem. J..

[13] Kocaoğlu Ç., Pediatric Intensive Care Unit, University of Health Sciences, Konya City Hospital, Konya, Turkey (2021). The Utility of Neutrophil Gelatinase-Associated Lipocalin in the Detection of Emerging Lung Injury Due to Mechanical Ventilation in Children: A Preliminary Study. Turk. Arch. Pediatr..

[14] Son E., Cho W.H., Jang J.H., Kim T., Jeon D., Kim Y.S., Yeo H.J. (2022). Neutrophil Gelatinase-Associated Lipocalin as a Prognostic Biomarker of Severe Acute Respiratory Distress Syndrome. Sci. Rep..

[15] Clinical Management of Severe Acute Respiratory Infection When Novel Coronavirus (nCoV) Infection Is Suspected. https://www.who.int/publications-detail-redirect/10665-332299.

[16] Grudzińska E., Grzegorczyn S., Czuba Z.P. (2019). Chemokines and Growth Factors Produced by Lymphocytes in the Incompetent Great Saphenous Vein. Mediat. Inflamm..

[17] Idzik M., Poloczek J., Skrzep-Poloczek B., Dróżdż E., Chełmecka E., Czuba Z., Jochem J., Stygar D. (2022). The Effects of 21-Day General Rehabilitation after Hip or Knee Surgical Implantation on Plasma Levels of Selected Interleukins, VEGF, TNF-α, PDGF-BB, and Eotaxin-1. Biomolecules.

[18] Gómez-Casado C., Roth-Walter F., Jensen-Jarolim E., Díaz-Perales A., Pacios L.F. (2013). Modeling Iron-Catecholates Binding to NGAL Protein. J. Mol. Graph. Model..

[19] Freund O., Breslavsky A., Givoli-Vilensky R., Zacks N., Gershman E., Melloul A., Wand O., Bilenko N., Bar-Shai A. (2023). Assessment of a Close Respiratory Follow-up Schedule at 3 and 6 Months after Acute COVID-19 and Its Related Investigations. Respir. Med..

[20] Kunnumakkara A.B., Rana V., Parama D., Banik K., Girisa S., Henamayee S., Thakur K.K., Dutta U., Garodia P., Gupta S.C. (2021). COVID-19, Cytokines, Inflammation, and Spices: How Are They Related?. Life Sci..

[21] Hazeldine J., Lord J.M. (2021). Neutrophils and COVID-19: Active Participants and Rational Therapeutic Targets. Front. Immunol..

[22] Abella V., Scotece M., Conde J., Gómez R., Lois A., Pino J., Gómez-Reino J.J., Lago F., Mobasheri A., Gualillo O. (2015). The Potential of Lipocalin-2/NGAL as Biomarker for Inflammatory and Metabolic Diseases. Biomarkers.

[23] Skrypnyk N.I., Gist K.M., Okamura K., Montford J.R., You Z., Yang H., Moldovan R., Bodoni E., Blaine J.T., Edelstein C.L. (2020). IL-6-Mediated Hepatocyte Production Is the Primary Source of Plasma and Urine Neutrophil Gelatinase–Associated Lipocalin during Acute Kidney Injury. Kidney Int..

[24] Chakraborty S., Kaur S., Guha S., Batra S.K. (2012). The Multifaceted Roles of Neutrophil Gelatinase Associated Lipocalin (NGAL) in Inflammation and Cancer. Biochim. Biophys. Acta (BBA) Rev. Cancer.

[25] Buonafine M., Martinez-Martinez E., Jaisser F. (2018). More than a Simple Biomarker: The Role of NGAL in Cardiovascular and Renal Diseases. Clin. Sci..

[26] Guo H., Jin D., Chen X. (2014). Lipocalin 2 Is a Regulator Of Macrophage Polarization and NF-ΚB/STAT3 Pathway Activation. Mol. Endocrinol..

[27] Baggiolini M., Clark-Lewis I. (1992). Interleukin-8, a Chemotactic and Inflammatory Cytokine. FEBS Lett..

[28] Kaiser R., Leunig A., Pekayvaz K., Popp O., Joppich M., Polewka V., Escaig R., Anjum A., Hoffknecht M.-L., Gold C. (2021). Self-Sustaining IL-8 Loops Drive a Prothrombotic Neutrophil Phenotype in Severe COVID-19. JCI Insight.

[29] Can E., Oğlak S.C., Ölmez F., Bulut H. (2022). Serum Neutrophil Gelatinase-Associated Lipocalin Concentrations Are Significantly Associated with the Severity of COVID-19 in Pregnant Patients. Saudi Med. J..

[30] Flemming A. (2022). Dexamethasone Restrains Neutrophils in Severe COVID-19. Nat. Rev. Immunol..

[31] Wang Y., Jia M., Yan X., Cao L., Barnes P.J., Adcock I.M., Huang M., Yao X. (2017). Increased Neutrophil Gelatinase-Associated Lipocalin (NGAL) Promotes Airway Remodelling in Chronic Obstructive Pulmonary Disease. Clin. Sci..

[32] Kawagoe J., Kono Y., Togashi Y., Ishiwari M., Toriyama K., Yajima C., Nakayama H., Kasagi S., Abe S., Setoguchi Y. (2021). Serum Neutrophil Gelatinase-Associated Lipocalin (NGAL) Is Elevated in Patients with Asthma and Airway Obstruction. Curr. Med. Sci..

[33] Hassler L., Wysocki J., Gelarden I., Sharma I., Tomatsidou A., Ye M., Gula H., Nicoleascu V., Randall G., Pshenychnyi S. (2022). A Novel Soluble ACE2 Protein Provides Lung and Kidney Protection in Mice Susceptible to Lethal SARS-CoV-2 Infection. J. Am. Soc. Nephrol..

